# Pilot study of positive airway pressure usage, patient journey and program engagement for users of a digital obstructive sleep apnea program

**DOI:** 10.3389/fdgth.2023.1043578

**Published:** 2023-06-07

**Authors:** Shefali Kumar, Emma Rudie, Cynthia Dorsey, Kimberly Caswell, Amy Blase, Fatima Sert Kuniyoshi, Adam V. Benjafield, Shannon S. Sullivan

**Affiliations:** ^1^ Verily Life Sciences, South San Francisco, CA, United States; ^2^ ResMed Science Center, San Diego, CA, United States

**Keywords:** obstructive sleep apnea management, remote care pathway, positive airway pressure, telemedicine, PAP

## Abstract

**Purpose:**

This single-arm, decentralized pilot study assessed patient journey, positive airway pressure (PAP) usage and program satisfaction for users of an entirely virtual telemedicine program for obstructive sleep apnea (OSA) diagnosis and management. This analysis focuses specifically on the subset of participants in the program who were diagnosed with OSA and prescribed PAP therapy.

**Methods:**

The Verily Clinical Studies Platform was used for virtual screening, consent, and enrolling eligible patients from North Carolina and Texas. After completing the virtual OSA diagnosis process, participants diagnosed with OSA and prescribed PAP therapy downloaded the program's mobile app. The app featured tools such as educational content, live coaching support, and motivational enhancement.

**Results:**

Of the patients included in this analysis (*N* = 105), the majority were female (58%), and white (90%). The mean time from first televisit to PAP initiation was 29.2 (SD 12.8) days and f 68 out of the 105 patients (65%) reached 90-day adherence. On average, patients used their PAP device for 4.4 h per day, and 5.4 h on days used. Engagement with the app was associated with higher rates of PAP adherence. Adherent individuals used the mobile app 52 out of the 90 days on average, compared to non-adherent individuals who used the app on 35 out of the 90 days on average (*p* = 0.0003).

**Conclusions:**

All of the 105 patients in this program diagnosed with OSA and prescribed PAP therapy were able to efficiently complete the entire OSA diagnostic pathway. The majority of these individuals also were able to adhere to their prescribed PAP therapy and had clinically meaningful PAP usage rates over the 90 days of therapy. Future studies might further evaluate the impact of this type of end-to-end virtual program on longer-term adherence and clinical outcomes over time.

**Clinical Trial Registration:**

https://clinicaltrials.gov/ct2/show/NCT04599803?term=NCT04599803&draw=2&rank=1, identifier NCT04599803.

## Introduction

Obstructive sleep apnea (OSA) impacts about one billion individuals worldwide. If untreated, it is associated with diminished quality of life, adverse health outcomes, and serious and costly chronic comorbidities (1, 2). Positive airway pressure (PAP) therapy is recommended as the first-line therapy option for OSA patients (3). Despite the importance and proven efficacy of PAP therapy, overall PAP adherence remains suboptimal; PAP adherence may settle between 25% and 83% (4–10). Hwang and colleagues reported on a telemedicine program for referred clinical patients demonstrating a 90-day PAP adherence rate of 58% (6). Studies have shown the importance of supporting PAP usage during the initial weeks of use and have suggested that PAP adherence is critical for achieving the clinical benefits and potential economic benefits associated with therapy (11–14).

The OSA diagnosis and treatment pathway, which may be complex, multi-step, fragmented and confusing, is a contributor to poor PAP therapy uptake (12). Potential barriers to achieving optimal PAP use may include discomfort with the mask or device, lack of continuous support throughout therapy and a lack of education and understanding about OSA and PAP therapy. Barriers to PAP adherence also can include individual patient factors such as disease severity, comorbidities, psychosocial support, and adherence related cognitions, such as beliefs about self-efficacy related to PAP use and expected positive outcomes from maintaining PAP therapy (15, 16).

Education and motivational enhancement, as well as telemedicine interventions, may improve early PAP adherence (7, 17). An increased interest in telemedicine and innovative methods of care has developed in recent years, augmented by the Covid-19 pandemic. Although virtual care methods are increasingly being used to diagnose and treat sleep disorders (18, 19), there are few supportive, end-to-end, virtual solutions that identify OSA risk and guide patients through the multi-step diagnosis and treatment process.

The feasibility study was designed to assess a comprehensive virtual program for OSA diagnosis and management. We previously reported evidence on patient journey metrics from the study for all participants (20), The objective of the current analysis is to provide insights into the PAP usage patterns and adherence to treatment for those patients, who were diagnosed with OSA within the virtual care pathway and prescribed and treated with PAP therapy.

## Methods

### Study design

This prospective, single-arm, decentralized pilot study aimed to assess the feasibility of a new, fully virtual program for assessing, diagnosing, and managing OSA. In order to allow for a broad study coverage area, while maintaining compliance with regulatory frameworks for virtual consent, and to accommodate appropriate medical licensure, recruitment for participation was extended to two states, North Carolina and Texas. Verily's Baseline Platform (now called Clinical Studies Platform), a comprehensive remote clinical studies platform, was used for study recruitment, consent, screening, enrollment, data collection, and study monitoring. The Clinical Studies Platform is Part 11 compliant and ISO 27001 certified to ensure compliance with leading security standards. The study is registered on clinicaltrials.gov (NCT04599803) and was approved by Western Institutional Review Board (Olympia, Washington). Full details on the study design have been published previously (20).

### Program overview

The platform is designed to help individuals who may have OSA to undergo screening questionnaires, schedule and complete televisits with board-certified sleep physicians, engage in interactive education modules, and coordinate home sleep apnea testing if this is ordered by the physician. For those diagnosed with OSA after testing and a second physician visit, if the physician prescribes PAP therapy, a mobile application is then used to support therapy setup and ongoing management. Communication with trained health coaches is available through the app for continuous additional support. All coaches were trained on a motivational enhancement approach that was adapted for asynchronous texting from a previously established protocol (21). It included reflection on personal goals, education, support for goal setting, exploration of ambivalence, and support for patient autonomy and self-efficacy. Coaches helped patients with identification of obstacles to PAP use and problem-solving strategies. Trained and experienced coaches delivered all texting with a motivational interviewing style of communication. When needed, coaches supported patients through phone calls. Escalated care to sleep physicians was also available as needed.

### Recruitment and study eligibility

Participants were recruited through an online registry (https://www.projectbaseline.com/) and digital advertisements. The main recruitment tool was a web landing page that provided a full study overview and the means to consent to the Project Baseline Community Study, an online registry for individuals interested in opportunities to participate in health-related research, test new technology, and learn about their health. Once enrolled in the Project Baseline Community Study. From this, participants could proceed to an online preassessment tool to assess initial eligibility. To be eligible, participants needed to own a compatible smartphone, have access to electricity, internet and a computer with a webcam, had to be at least 18 years old, speak and read English, and reside in North Carolina or Texas. Participants also had to score 5 or greater (high risk for OSA) on the OSA-50 questionnaire (22). The exclusion criteria included a previous diagnosis of sleep apnea, chronic insomnia, shift work, pregnancy or a plan to become pregnant during the study, current use of supplemental oxygen, or employment by the sponsor.

To be eligible for the PAP therapy portion of the study, participants had to have undergone sleep testing and to have subsequently been diagnosed with OSA by a board certified sleep physician. Also, participants were only eligible if they had been prescribed PAP by their sleep physician following a 30-min visit during which results, diagnosis-related information, and treatment options were discussed. Participants who did not complete the home sleep apnea test (HSAT) by December 31, 2020 were not eligible for continued participation. In addition, when other evaluations or therapies were recommended by the sleep physician, participants were off-boarded from the study with efforts made to connect them to appropriate next steps.

### Procedures

Potential participants learned the details about the study on the study website. After completing the online screener, which included medical and sleep history as well as completion of the Epworth Sleepiness Scale (ESS), those eligible and enrolled scheduled their first televisit with a sleep physician. During the visit, the physician determined whether an HSAT was an appropriate next step, based on their clinical judgment. If HSAT was recommended, patients were directly sent their HSAT equipment (WatchPat One, Itamar, Caesarea, Israel). The Itamar WatchPAT ONE device utilizes peripheral arterial tonometry (PAT) for OSA diagnosis. It measures 7 channels (PAT® signal, heart rate, oximetry, actigraphy, body position, snoring, and chest motion) via three points of contact. The device has been clinically validated with high correlation between respiratory indices calculated using PAT vs. polysomnography (PSG) and a statistically significant correlation between Watch PAT-determined apnea hypopnea index (AHI) scores and PSG AHI scores (Spearman's rho = 0.802 *p* < 0.001) (23, 24).

Upon completion of the HSAT, results were uploaded and participants scheduled a second televisit with their physician. The sleep physician, upon review of the the HSAT results, combined with their clinical judgment, made a diagnosis, which was discussed with patients together with treatment options.

If PAP therapy was recommended, participants downloaded the mobile app (full description of the app capabilities can be found in the initial report of this study 20). As part of the onboarding process, participants logged in the app their key symptoms, how their symptoms impacted their functioning and quality of life, and their personal treatment goals. Participants selected a mask and ordered their PAP device and supplies (AirSense 10, ResMed, San Diego, CA) through the app.

The app guided participants through PAP set-up, practice with mask and device, and ongoing PAP use. This support included an audio-guided brief daytime practice session with PAP and audio-guided relaxing imagery at the beginning of the initial night and first week of PAP use. Participants could reach out to their coaches as needed throughout the study.

Five study surveys were administered online to assess demographics, OSA knowledge, and program satisfaction. A final televisit was conducted at the end of 90 days of therapy, during which participants could discuss continued OSA care and PAP therapy (if desired) outside of the study.

### Outcome measures

The analysis presented in this manuscript focuses on the cohort of participants who were prescribed PAP therapy. Metrics included PAP usage, program engagement, and program satisfaction. In addition, as journey times through the assessment pathway are reported elsewhere, the number of days from the first televisit to initiating therapy was assessed for this analysis.

PAP adherence is defined as a dichotomous variable where a participant is considered adherent if they use their device at least 4 h per day on at least 21 days out of a consecutive 30-day period within the first 90 days of PAP therapy, per Centers for Medicare and Medicaid Services guidelines (25). PAP usage was assessed by calculating the following metrics:

1) Number of days the device was used.
2) Average PAP use per participant, defined as the total number of hours a participant used the PAP device during the 90-day study, divided by 90 days.
3) Average therapy session length, defined as the total number of hours a participant used the PAP device during the 90-day study, divided by the count of days on which that participant actually used the device.

Data on PAP usage was collected via a cloud-based platform (AirView, ResMed, San Diego, CA). For all metrics, mean, median, standard deviation, and interquartile range (IQR) are reported for the overall cohort, and for adherent and non-adherent participants. Data for individuals who withdrew during the PAP therapy period are included in the analysis with zero usage assumed for days post-withdrawal.

Program engagement was assessed by the number of days the app was opened. Coaching engagement was based on the number of in-app messages sent by a participant to the coach. Participants who sent more than 3 messages were categorized as “coaching users.” This threshold was applied because the first few messages sent were typically introductory.

Program satisfaction was measured using a standard five-point Likert scale ranging from “very dissatisfied” (1) to “very satisfied” (5).

### Sample size and statistical analyses

The study was a feasibility pilot. We exceeded our target of studying at least 50 individuals on PAP therapy. Standard power analyses were conducted in Python 3 using the scipy stats module, assuming 95% confidence and 80% power, and calculated over a range of plausible standard deviations.

We used Chi-squared tests to compare several journey components across different subcohorts of interest: to determine if the frequency of adherence was different among those with vs. without each of the common comorbidities, evaluating each comorbidity independently; to evaluate whether the engagement with the coach (reflected by the number of messages sent) affected adherence, evaluating frequency of adherence among 2 groups: those who sent at least 4 messages to their coach during the study vs. those who sent fewer than 4; to determine whether the frequency of adherence was different among those who completed the satisfaction survey vs. those who did not; and to determine whether the frequency of adherence was different among participants who changed masks various number of times as compared to participants who changed masks fewer or more times (various groupings were used). To assess the strength of the relationship between app use and PAP use, we used linear regression, regressing number of days of app use for each participant on average hours of PAP use, reporting adjusted R-squared values.

## Results

### Participant demographics and clinical characteristics

**Figure 1:**
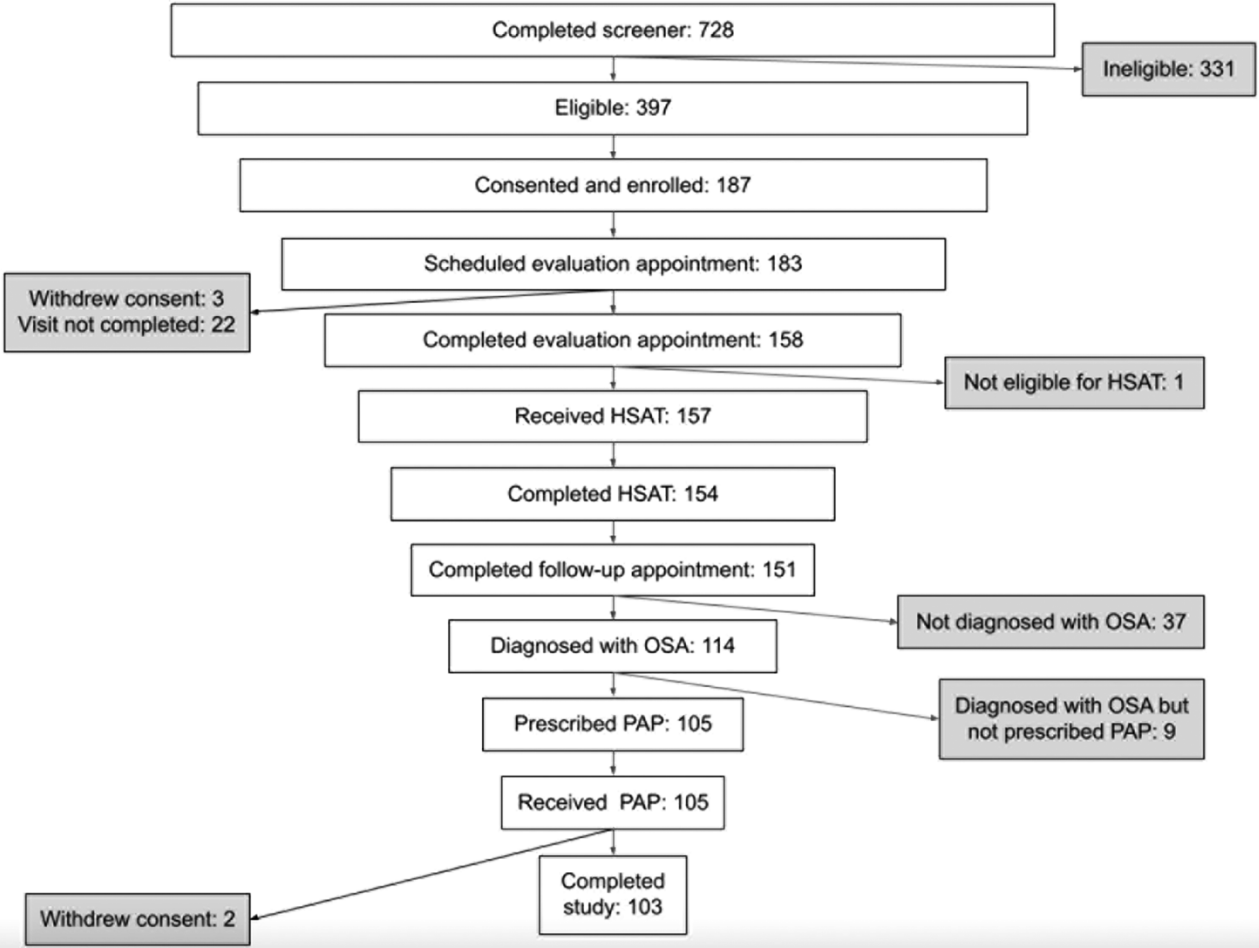
Consort diagram showing number of participants reaching each stage of the program.

A total of 105 individuals were prescribed PAP therapy through the program (Figure 1). Two participants withdrew from the study during the 90-day therapy period (withdrew days 43 and 84) for no specified reason. The mean and median age of this cohort were both 48 years with the majority of the population under the age of 50. The majority of the population was female (58%), and white (90%). A total of 53% of participants reported a baseline body mass index (BMI) ≥ 35 kg/m^2^. Baseline ESS was 11.3 (SD 4.8). The most commonly reported comorbidities were allergies (50%), high blood pressure (40%), and diabetes (18%). Table 1 provides a summary of key demographics, comorbidity and clinical characteristics.

**Table 1:** Key baseline demographic and self-reported clinical characteristics of population who initiated PAP therapy (*n* = 105).

Characteristics		Value
Age (years), mean (SD)		48.0 (10.3)
Sex, *n* (%)	Female	61 (58)
	Male	44 (42)
Race/ethnicity, *n* (%)*	White	94 (90)
	Black	3 (3)
	Hispanic	25 (24)
	Asian	2 (2)
	Native American	4 (4)
	Other	6 (6)
Geographic Location, *n* (%)	North Carolina	43 (41)
	Texas	62 (59)
Comorbidities, *n* (%)	Allergies	52 (50)
	High blood pressure	42 (40)
	Diabetes	19 (18)
	None	17 (16)
	Swollen legs	14 (13)
	Bronchitis or asthma	14 (13)
	Migraine	13 (12)
	Thyroid problems	12 (11)
	Bruxism	12 (11)
	Anemia	9 (9)
	Dizziness or fainting	8 (8)
	Chronic pain	8 (8)
	Heart murmur	7 (7)
	Arrhythmia	6 (6)
	Other	6 (6)
	Concussion	5 (5)
	Mononucleosis	5 (5)
	Coronary artery disease	5 (5)
	TMJ	4 (4)
	Heart attack	3 (3)
	COPD	3 (3)
	Strokes	2 (2)
	Epilepsy or seizures	2 (2)
	Heart failure	1 (1)
BMI, mean (SD) kg/m^2^		36.5 (8.7)
BMI, *n* (%)	<18.5 kg/m^2^	0 (0)
	18.5–<25 kg/m^2^	6 (6)
	25–<30 kg/m^2^	19 (18)
	30–<35 kg/m^2^	24 (23)
	35 + kg/m^2^	56 (53)
OSA-50, mean (SD)		7.4 (1.6)
ESS, mean (SD)		11.3 (4.8)
ESS, *n* (%)	0–5	14 (13)
	6–10	34 (32)
	11–12	11 (10)
	13–15	29 (28)
	16–24	17 (16)
pAHI, mean (SD)		23.3 (24)
pAHI, *n* (%)	<5	14 (13)
	5–14	37 (35)
	15–29	29 (28)
	30+	25 (24)

BMI, body mass index; ESS, epworth sleepiness scale; OSA, obstructive sleep apnea; SD, standard deviation; pAHI, apnea-hypopnea index by peripheral arterial tonometry device; TMJ, temporomandibular disorders.

* Does not sum to 100% because participants were able to select multiple answer choices.

### OSA screening and HSAT results

Average OSA-50 score was 7.4 points and average apnea-hypopnea index determined by peripheral arterial tonometry devices (pAHI) at diagnosis was 23.3 (SD 23.8) events per hour using a 4% desaturation threshold. A total of 13% had a pAHI < 5; mean pAHI in this group was 2.3 (SD 1.4). Additionally, 35% had pAHI 5–14.9, 28% had pAHI 15–29.9, and 24% participants had pAHI ≥ 30.

### Patient journey metrics

In those diagnosed with OSA, the mean interval from first televisit to PAP initiation was 29.2 (SD 12.8) days, with a median of 25 days (Table 2). In subgroup analysis based on adherence, mean interval was 28.2 (SD 13.7) days for adherent participants and 31.0 (SD 11.0) days for non-adherent participants (*p* = 0.25).

**Table 2:** Key metrics overall and by adherence.

	Overall (*N* = 105)		Adherent (*n* = 68)		Non-adherent (*n* = 37)	
	mean (SD)	median (IQR)	mean (SD)	median (IQR)	mean (SD)	median (IQR)
**Time from 1st physician visit to (days):**						
2nd physician visit	20.7 (9.3)	18 (15, 24)	19.2 (8.3)	17.5 (14.75, 21.25)	23.5 (10.4)	21 (16, 27)
To HSAT being received by patient	7.0 (3.0)	7 (6, 8)	7.1 (3.4)	7 (6, 7.25)	6.8 (2.2)	6 (6, 8)
Therapy initiation	29.2 (12.8)	25 (22, 33)	28.2 (13.6)	24.5 (22, 29.25)	31.0 (10.9)	28 (22, 40)
**PAP use:**						
Days	67.5 (25.5)	79 (54, 87)	79.8 (13.0)	84.5 (77, 89)	43.6 (26.7)	48 (19, 66)
Average PAP use (hours)	4.4 (2.4)	4.4 (2.3, 6.3)	5.7 (1.6)	5.8 (4.4, 6.9)	1.7 (1.3)	1.2 (0.6, 2.4)
Average therapy session length (hours)	5.4 (1.9)	5.6 (4.2, 6.9)	6.4 (1.2)	6.4 (5.6, 7.2)	3.5 (1.6)	3.7 (2.0, 4.2)
**Coaching use:**						
Participants with >3 messages (%)	92		97		84	

HSAT, home sleep apnea test; IQR, inter-quartile range; SD, standard deviation.

### PAP usage

**Figure 2:**
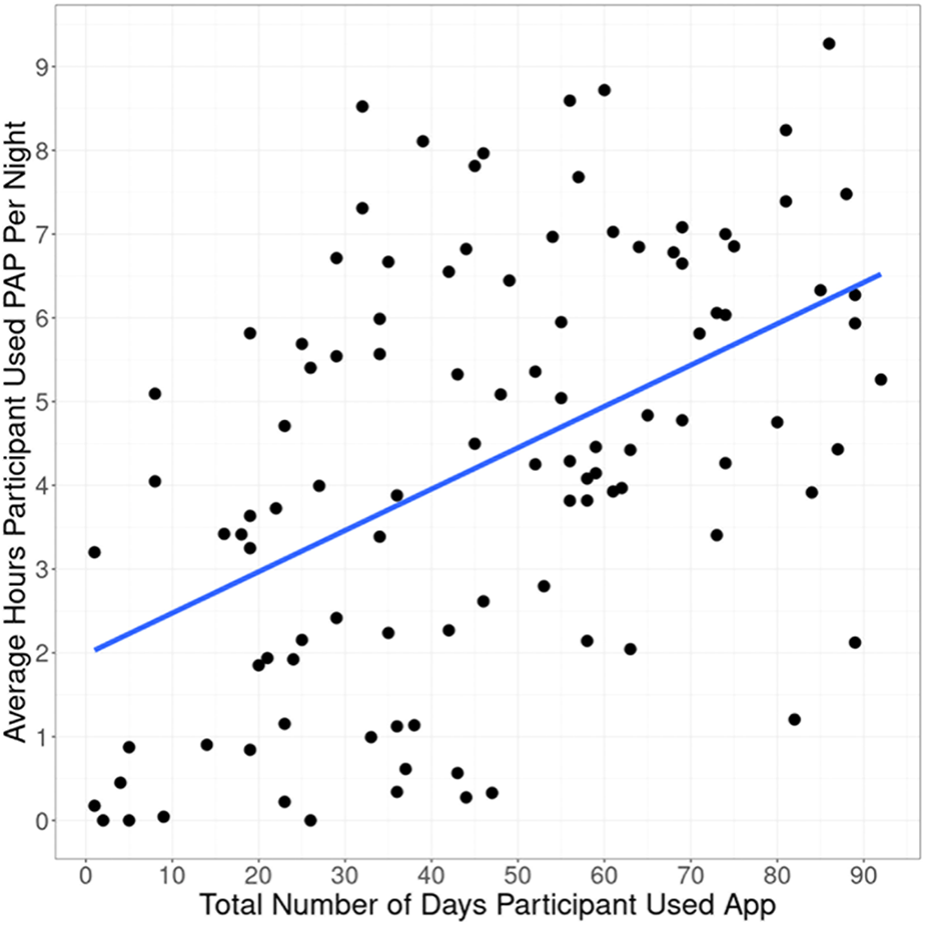
Average PAP use vs. Total days of app use.

Of the participants prescribed PAP therapy, 65% achieved 90-day adherence. It took on average 37.5 days from therapy initiation to reach adherence. On average, participants used the PAP device on 75% of days (Table 2). Average PAP use per participant was 4.4 h, and average therapy session length was 5.4 h (Figure 2). Only 3 participants (3%) did not use the PAP device during the study period. Overall, 86% used the device for at least 30 days, 70% used the device for at least 60 days, and 7% used the device on all 90 days.

**Figure 3:**
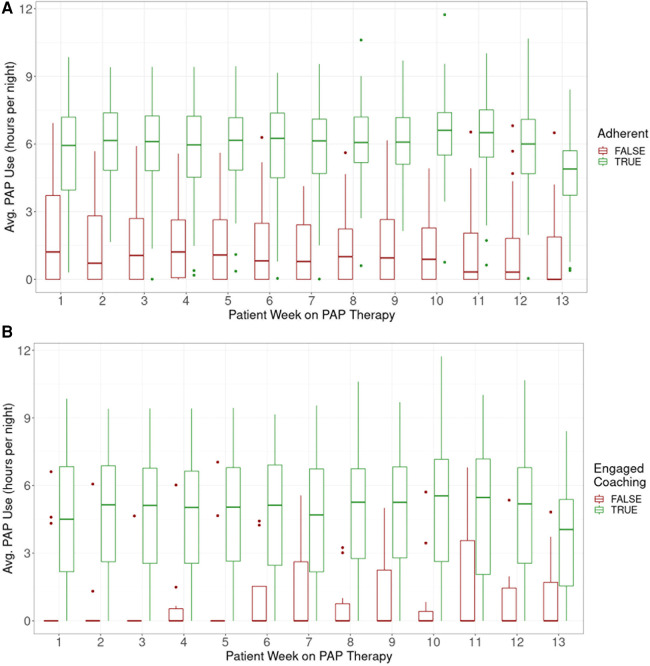
Average PAP use per participant by week of therapy, (**A**) adherent vs. Non-adherent, (**B**) by coach use (‘use’ defined as >3 messages sent by participant).

Although average usage was consistently higher among adherent participants based on CMS criteria for adherence, non-adherent participants as a group had PAP usage which continued through the initiation of study end activities (Figure 3A).

**Figure 4:**
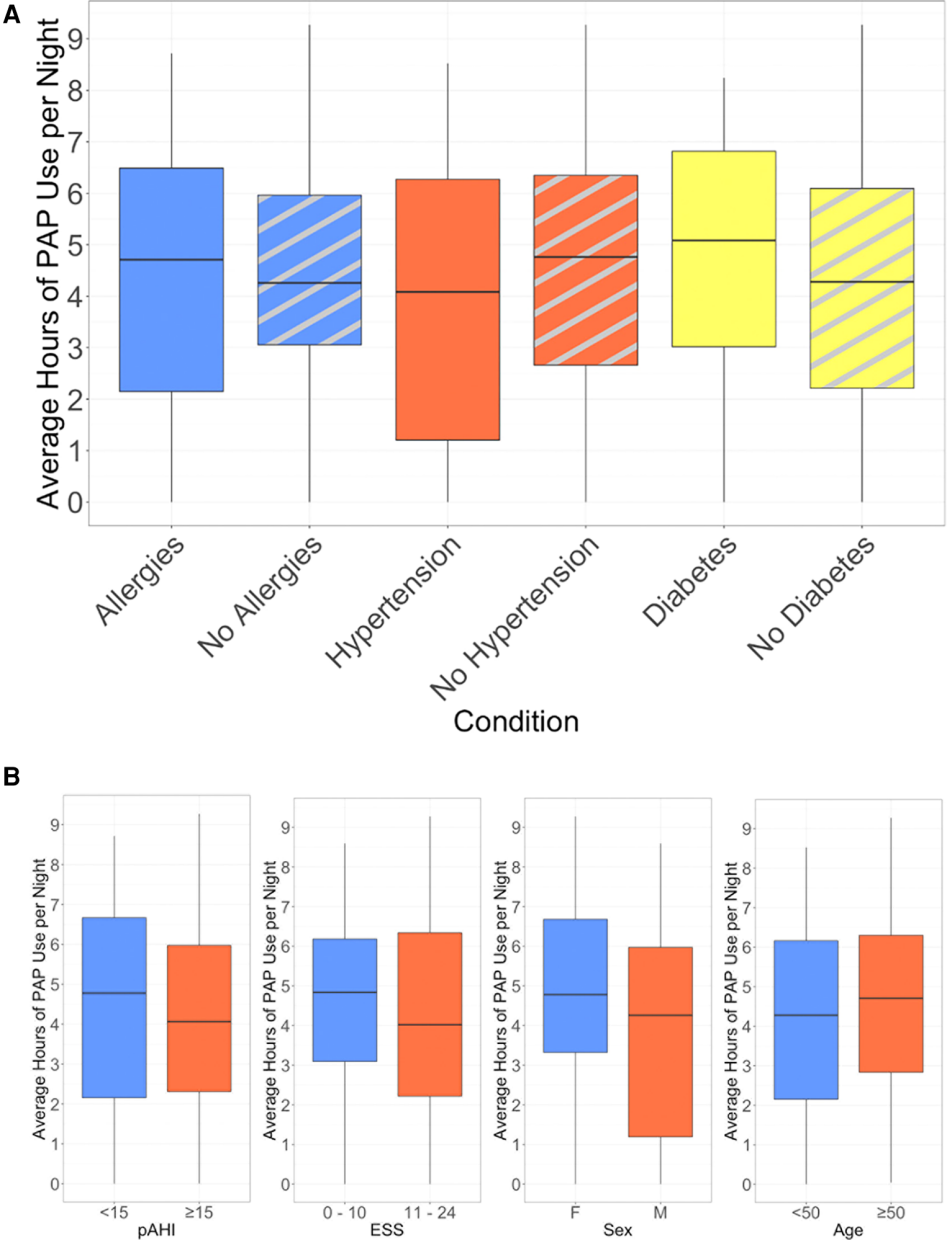
Average hours of nightly PAP use, (**A**) stratified by presence of selected comorbidities, (**B**) stratified by baseline characteristics.

Using the top three most common comorbidities reported in this group, we evaluated whether these comorbidities were associated with PAP usage (Figure 4A). We also assessed whether age group, sex, initial ESS, or pAHI had an impact on PAP usage (Figure 4B). There was no association between 90-day PAP usage and these demographic, disease, or comorbidity characteristics.

### Mobile app and coach engagement

Engagement with the mobile app was associated with higher PAP adherence rates. The adjusted R-squared from regressing days of app use on average PAP use was 0.225 (*p* < 0.0001) (Figure 2). On average, participants used the app 46 out of 90 days. Adherent individuals used the app for 52 days on average; non-adherent individuals used the app for 35 days on average (*p* = 0.0003).

Engagement with a coach was also associated with higher adherence rates. Overall, 95% of participants sent at least one in-app message to a coach and 92% sent at least 4 messages. Of those who sent at least 4 messages, 68% were adherent; of those who sent fewer than 4 messages, 25% were adherent (*p* = 0.02, Figure 3B).

### Program satisfaction

Overall average program satisfaction at study end was 4.4 out of 5 (*n* = 74), with 58% (*n* = 43) of respondents rating their satisfaction 5 out of 5. Only 4% (*n* = 3) of respondents rated their satisfaction negatively (1 or 2). Of the 62 individuals who rated their experience positively (4 or 5), 45 were adherent (73%). Adherence was lowest for those who did not complete the survey (*n* = 29), at 45% (*p* = 0.02).

### Mask changes

Overall, 69% of participants changed masks at least once during the study period, with 34% changing masks once, 21% changing masks twice, and 13% changing masks more than twice. Adherence was 55% among participants who never changed masks, 78% among participants who exchanged their mask once, and 61% among those who changed masks more than once (*p* = 0.14). Comparing adherence of those who changed exactly once to all others was borderline statistically significant (*p* = 0.05).

## Discussion

Overall, study participants efficiently completed the diagnostic pathway and those who were prescribed PAP therapy initiated it effectively. On average, the interval from initial televisit for evaluation to PAP therapy initiation was less than one month. The majority (65%) of participants who initiated PAP therapy reached 90-day adherence based on CMS criteria, and those who were adherent used PAP for an average of 5.4 h on days used. For comparison, reported adherence rates vary greatly in the United States, with some estimates of CMS-defined adherence from meta-analyses between 25 and 83% (4–10). A sleep telemedicine program for OSA has reported an adherence rate of 58% (7, 15, 16); and another recent real-world report of a fully-remote diagnostic, treatment, and monitoring care for OSA patients demonstrated CMS 90-day adherence of 41% (8).

This study was not designed for direct comparison to PAP adherence and usage of traditional clinical pathways, as the study population likely differs substantially from those who experience a traditional OSA diagnostic pathway. This population was recruited from outside the typical clinical environment, on the internet. This may have introduced bias towards participants who were less motivated to seek treatment and manage their OSA than those who are physician-referred or who seek in-person diagnosis and treatment on their own. Nonetheless, these results are promising; they suggest that individuals identified via novel methods who complete a virtual OSA diagnostic and treatment pathway may have comparable adherence rates and PAP usage to patients who are diagnosed with OSA in-person and onboarded onto therapy using traditional care delivery methods.

This pilot evidence indicates that such fully virtual approaches, when applied with adequate interactions with board-certified sleep practitioners and evidence-based support from coaches, could be a scalable and efficient solution to diagnose and treat individuals with undiagnosed OSA. In our study, only 3 individuals completely stopped treatment after trying PAP. This is a promising finding, in comparison to estimates from other studies suggesting that 8%–15% stop after the first night of treatment (4, 26). In this study, even among non-adherent users, PAP usage persisted with a pattern of regular use through about week 10 when study off-ramping activities were typically initiated. Thus we believe that there may be an opportunity for continued support to achieve ultimate success for those who may be considered “noncompliant” by traditional standards. Another important finding from this pilot is that participant age group, sex, comorbid condition, pAHI severity, and initial ESS did not have a significant impact on PAP adherence at 90 days. This is a novel finding for a fully remote pathway, and starts to provide evidence that technology-enabled care pathways have the possibility to be effective for a wide variety of participants without requiring extensive patient pre-selection.

Program satisfaction and app usage was also quite high. Participants used the mobile app more than 50% of days on average, and more than 90% of patients actively messaged their coach for support. Both program engagement and actively messaging the coach were positively correlated with PAP adherence. Even when including those who were not adherent, overall mobile app use was directionally correlated with hours of PAP use. The underlying motivational enhancement approach elements and accompanying coach support may have promoted adherence.

It has been established that a poor-fitting mask can contribute to a lack of motivation to use PAP therapy, resulting in low adherence or rejection of therapy (27). Participants with one mask change in the study had the highest rate of adherence. This could possibly be a result of starting PAP therapy in a virtual setting, and could be important to incorporate in remote therapy programs. Initiating and adhering to PAP therapy also involves behavior change, and self-efficacy about treatment is a consistent predictor of PAP therapy adherence (16) Participants may have experienced a sense of control and self-efficacy when given the option and support to easily change their mask to best fit their needs. The ability to make one change may be ideal, with diminishing returns on subsequent changes.

Overall, these study results are encouraging. Participants had relatively high adherence and PAP usage despite going through a novel care delivery method. Given recent challenges and changes to traditional models of care accelerated by the Covid-19 pandemic, as well as ensuing coverage policy changes expanding access to telehealth care, an end-to-end virtual platform may be a valuable mechanism to provide effective, responsible, patient-centered care pathways for those with OSA.

### Strengths and limitations

Key strengths and limitations of the study design have been previously published (20). One key strength and potential benefit of real-world application of this program is that it was conducted fully virtually. Another strength is that the analyses reflect real world circumstances. For example, participants diagnosed with covid-19 were asked not to use their PAP device for 10 days. Some participants were also impacted by the February 2021 weather-related widespread power outages in Texas and were unable to use their PAP device. These data still were included in all analyses.

This study had some limitations. Since this was a single-arm pilot study, there was no comparison control group. It would have been difficult to construct a true generalizable control arm since the program included diagnosing participants with OSA, inherently making them different from a generalizable population. pAHI was also not part of PAP therapy eligibility criteria; in some settings, this may limit generalizability, but was an intentional part of the study design. OSA diagnosis, assessment of symptoms, and decisions regarding treatment options, were all left to the study physicians. Among those on PAP therapy, almost half had pAHI scores less than 15.0 events per hour, and 13% had a pAHI less than or equal to 5.0 events per hour. While HSATs are known to be insensitive to certain presentations of OSA, in real-world settings, patients with a pAHI less than 5.0 may have been referred for in-lab testing.

Also unlike standard clinical practice, participants were not incentivized, under risk of paying out-of-pocket or returning their device, to reach 90-day adherence. The program had no messaging around payor requirements, but rather focused on education, personalized support, and motivational techniques. Participants received a free PAP device with supplies, and were able to keep their device once the study had ended irrespective of whether they used their device or not. It is plausible that with no financial consequences at stake, participants were less motivated to use their device and reach 90-day adherence criteria. Therefore, the adherence rate of 65% may under-represent the full potential of this sort of program if deployed broadly in the real world. Finally, the generalizability of our results may require examination in subsequent studies, since the cohort of patients included in this analysis consisted of mostly White female patients.

## Conclusions

These findings show that a cohort of patients using this end-to-end remote care platform were not only able to efficiently complete the entire OSA diagnostic pathway, but most also were able to adhere to PAP therapy. These findings are meaningful as they suggest that patients who experience a non-traditional entirely virtual diagnostic and treatment pathway can be successfully diagnosed and treated with PAP therapy and have PAP usage and adherence rates comparable to standard care when supported by evidence-based methods such as motivational enhancement to enhance adherence. Both increased engagement with the mobile app and a positive patient experience were found to be associated with increased adherence. Future studies might examine the relative impact of such elements in larger patient populations, and aim to better understand the impact of this type of virtual end-to-end platform on long-term adherence and clinical outcomes.

## Data Availability

Participants of this study did not agree for their data to be shared publicly for open use research, so supporting data is not available. Further inquiries can be directed to the corresponding author/s.
